# Disparities experienced by Aboriginal compared to non-Aboriginal metropolitan Western Australians in receiving coronary angiography following acute ischaemic heart disease: the impact of age and comorbidities

**DOI:** 10.1186/s12939-014-0093-3

**Published:** 2014-10-21

**Authors:** Derrick Lopez, Judith M Katzenellenbogen, Frank M Sanfilippo, John A Woods, Michael S T Hobbs, Matthew W Knuiman, Tom G Briffa, Peter L Thompson, Sandra C Thompson

**Affiliations:** Western Australian Centre for Rural Health, The University of Western Australia, Crawley, Western Australia Australia; School of Population Health, The University of Western Australia, Crawley, Western Australia Australia; Heart Research Institute, School of Medicine and Pharmacology, The University of Western Australia, Crawley, Western Australia Australia

**Keywords:** Aboriginal, Oceanic ancestry group, Australia, Ischaemic heart disease, Myocardial infarction, Healthcare Disparities, Hospitals, urban, Coronary angiography, Age factors, Comorbidity

## Abstract

**Introduction:**

Aboriginal Australians have a substantially higher frequency of ischaemic heart disease (IHD) events than their non-Aboriginal counterparts, together with a higher prevalence of comorbidities. The pattern of health service provision for IHD suggests inequitable delivery of important diagnostic procedures. Published data on disparities in IHD management among Aboriginal Australians are conflicting, and the role of comorbidities has not been adequately delineated. We compared the profiles of Aboriginal and non-Aboriginal patients in the metropolitan area undergoing emergency IHD admissions at Western Australian metropolitan hospitals, and investigated the determinants of receiving coronary angiography.

**Methods:**

Person-linked administrative hospital and mortality records were used to identify 28-day survivors of IHD emergency admission events (n =20,816) commencing at metropolitan hospitals in 2005–09. The outcome measure was receipt of angiography. The Aboriginal to non-Aboriginal risk ratio (RR) was estimated from a multivariable Poisson log-linear regression model with allowance for multiple IHD events in individuals. The subgroup of myocardial infarction (MI) events was modelled separately.

**Results:**

Compared with their non-Aboriginal counterparts, Aboriginal IHD patients were younger and more likely to have comorbidities. In the age- and sex-adjusted model, Aboriginal patients were less likely than others to receive angiography (RR_IHD_ 0.77, 95% CI 0.72-0.83; RR_MI_ 0.81, 95% CI 0.75-0.87) but in the full multivariable model this disparity was accounted for by comorbidities as well as IHD category and MI subtype, and private health insurance (RR_IHD_ 0.95, 95% CI 0.89-1.01; RR_MI_ 0.94, 95% CI 0.88-1.01). When stratified by age groups, this disparity was not significant in the 25–54 year age group (RR_MI_ 0.95, 95% CI 0.88-1.02) but was significant in the 55–84 year age group (RR_MI_ 0.88, 95% CI 0.77-0.99).

**Conclusions:**

The disproportionate under-management of older Aboriginal IHD patients is of particular concern. Regardless of age, the disparity between Aboriginal and non-Aboriginal Australians in receiving angiography for acute IHD in a metropolitan setting is mediated substantially by comorbidities. This constellation of health problems is a ‘double-whammy’ for Aboriginal people, predisposing them to IHD and also adversely impacting on their receipt of angiography. Further research should investigate how older age and comorbidities influence clinical decision making in this context.

## Introduction

Ischaemic heart disease (IHD) is the highest ranking contributor to the substantial life expectancy gap between Aboriginal and non-Aboriginal populations in Australia [1]. Coupled with this, Aboriginal Australians with IHD are known to have a higher prevalence of several major comorbid conditions that adversely influence outcomes [2] and may diminish the likelihood of receiving coronary angiography [3]. Coronary angiography is an integral component of guideline-adherent care for acute IHD and an essential precursor to a coronary artery revascularisation procedure (CARP). Disparity between Aboriginal and non-Aboriginal patients in receiving a CARP following acute admission for IHD has been explored in a several large cohort studies, with inconsistent findings [4-7].

This aim of this administrative linked data study was to investigate disparities in provision of coronary angiography to Aboriginal and non-Aboriginal IHD patients undergoing emergency admissions to metropolitan hospitals in Western Australia (WA). WA’s only metropolitan area (encompassing the capital city, Perth) had a population of around 1.6 million in 2011, of whom approximately 1.6% identify as Aboriginal [8]. The outcome examined was coronary angiography rather than CARP *per se*, as provision of CARP is dependent on patients’ angiographic findings, information which is not available from administrative records. We have previously reported on the rural population [9] and in this current paper we considered only the metropolitan population, as their pathways to receiving coronary diagnostic and intervention procedures are different from those of non-metropolitan patients. In WA, IHD patients from outside the metropolitan area require transfer to a cardiac catheterisation-capable hospital, the determinants of which can be complex [9-11]. There is also a complex relationship between age, sex, Aboriginal status, geographical residence (metropolitan, regional, very remote) and the incidence of myocardial infarction (MI), with non-metropolitan people not uniformly disadvantaged [12]. Compared with metropolitan people, regional Aboriginal men and very remote non-Aboriginal men aged 25–54 years have significantly higher incidence rates of MI. Furthermore, this paper addresses the sparse data about the health of Aboriginal people who live in metropolitan areas [13]. We separately analysed the subgroup of patients with MI, allowing investigation of a relatively homogeneous group for which diagnostic categorisation in administrative data is relatively accurate and for which there are well-defined evidence-based clinical guidelines for diagnosis and therapy.

## Methods

### Data source

A person-linked file of all metropolitan residents admitted to metropolitan hospitals from 2005 to 2009 with a principal discharge diagnostic code of IHD, incorporating their previous (15-year history) and subsequent hospital admissions, was extracted from the Hospital Morbidity Data Collection (HMDC) and Deaths datasets of the Western Australian Data Linkage System (WADLS). The WADLS is a comprehensive system linking population-based administrative health data from several statutory datasets through probabilistic matching, with the proportions of invalid (false positives) or missed links (false negatives) both estimated at 0.1% of matches [14].

### Study cohort

**Figure 1:**
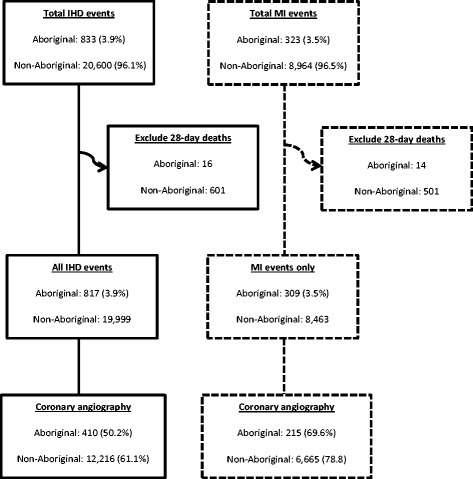
**Flow diagram of IHD and MI events from 2005–09 in metropolitan WA by Aboriginal status.** IHD = ischaemic heart disease; MI = myocardial infarction.

We identified IHD events in metropolitan residents aged 25–84 years who were admitted to metropolitan public and private hospitals from 2005 to 2009 (Figure 1). The starting point of an episode (defined as a series of contiguous hospital admissions, including inter-hospital transfers) was an emergency admission to a metropolitan hospital with a principal discharge diagnosis of IHD. An event included all admissions (booked or emergency) associated with the initial episode of care and any additional episodes starting within a 28-day period following the initial emergency admission. Any subsequent emergency IHD admission to metropolitan hospitals outside this event definition was considered a new event. Thus, a person could have multiple events over the study period. Events followed by death within 28 days were excluded, as it reduces the opportunity to receive coronary artery procedures. Sensitivity analyses were performed comparing outcomes with and without the 28-day deaths.

For each event, patient demographic variables and 15-year admission histories of specified comorbidities (chronic pulmonary disease, diabetes, heart failure [HF], kidney disease and pre-existing IHD) were recorded. Comorbidities were identified using International Classification of Diseases Australian Modification 10th revision (ICD-10-AM) codes defined by Quan et al. [15]. These comorbidities were selected as they are associated with increased risk of complications with coronary procedures [16,17]. The accuracy of identifying diabetes and HF in these datasets has been reported previously and can be improved by extending the hospitalisation look-back [18,19]. Sensitivity analyses were performed using comorbidities based on 1-, 2-, 5- and 10-year look-back periods. IHD category was classified as MI (ICD-10-AM: I21), unstable angina (I20.0) or other IHD (all other ICD codes between I20-I25) in the principal diagnosis field. MI subtype was categorised in line with ICD codes, namely transmural (I21.0-I21.3), subendocardial (I21.4) or other (I21.9), rather than by guidelines based on the presence or absence of electrocardiographic ST elevation. Socio-Economic Indexes for Areas (SEIFA) Index of Relative Socio-Economic Disadvantage scores categorised by quartiles were used as a measure of socio-economic status (SES) [20]. SEIFA scores are general population area-based measures of SES derived for each census Collection District (about 225 dwellings) [21]. For all events, SEIFA scores for a more aggregated geographical level (i.e. Statistical Local Area of residence) were used as some patients did not have a score at the Collection District level. As Aboriginal status is known to be under-reported in administrative health data [22,23], any patient identified as Aboriginal and/or Torres Strait Islander on any hospital admission since 1980 in the HMDC was classified as Aboriginal. Sensitivity analyses were performed for Aboriginal status based on (i) identification in at least 25% of hospital admissions (HMDC) and, (ii) identification at the initial admission for the event. Private health insurance status was defined as having private insurance at any admission during the event, as recorded in the HMDC. Australia has a publicly-funded universal healthcare system that aims to provide all Australians, regardless of personal circumstances, access to adequate care at an affordable or no cost to the individual. This is supported by optional private health insurance for hospital treatment as a private patient and for ancillary health services (e.g. physiotherapy) provided outside a hospital.

### Study outcomes

The outcome examined was coronary angiography. For each event, we determined if the patient received coronary angiography (Australian Classification of Health Interventions Block 668) within 28 days of the event admission date. Angiography was also assumed to have been performed if the patient had a CARP (Blocks 669–679) recorded within 28 days of the event admission date where angiography was not separately recorded in the procedure fields. Sensitivity analyses were performed comparing outcomes (receipt of coronary angiography among those surviving) for events defined by a 90-day instead of a 28-day interval, in order to investigate the effects of unexpected delays in cardiac catheterisation.

### Statistical analyses

Analyses were performed using SAS 9.4 (SAS Institute). Baseline demographic and comorbidity characteristics of events involving Aboriginal and non-Aboriginal subjects were summarised separately. Chi-square tests were used to test for significance of categorical variables. To model event-based receipt of coronary angiography allowing for multiple events in each patient, a Poisson distribution for the dependent variable, together with a log link function, exchangeable variance structure across multiple events for a patient, maximum-likelihood method and robust standard errors was used. When the outcome is binary, the exponentiated coefficients from the Poisson regression represent risk ratios (RR) rather than incidence-rate ratios and the robust standard errors take into account both the use of the Poisson model as a working model for binary data and the clustering of events within individuals [24]. In addition to an unadjusted model that included Aboriginal status only, six models with sequential addition of variables were developed: Model 1 (10-year age categories and sex); Model 2 (Model 1 + SES); Model 3 (Model 2 + private health insurance); Model 4 (Model 3 + IHD category/MI subtype and coronary angiography in the last year); Model 5 versions a-e (Model 4 + individual comorbidities); and Model 6 (Model 4 + all comorbidities combined). Although data on type of hospital (tertiary, other public, private) were available in the HMDS, we did not include the hospital type at initial admission in the full model given the complex determinants of hospital of initial admission for acute IHD; this includes results of pre-hospital (ambulance-based) electrocardiographic testing [25], time of day, and traffic conditions. Furthermore, metropolitan IHD patients are commonly transferred between hospitals for treatment. Sensitivity analysis based on hospital type at initial admission is presented. Previous work from our group has identified different age and sex distribution of incident MI cases in Aboriginal and non-Aboriginal people [26]. Accordingly, interactions between Aboriginal status*age and Aboriginal status*sex were investigated. In whole cohort analyses age was modelled using 10-year age categories but in the broad age group specific analyses (25–54 and 55–84 years) age was modelled using age and age*age.

### Ethics

Ethics approval was obtained from Human Research Ethics Committees of WA Aboriginal Health, The University of Western Australia and WA Department of Health.

## Results

### Event characteristics

Of the 21,433 IHD events identified in metropolitan hospitals from 2005 to 2009 (Figure 1), 20,816 (97.1%) resulted in survival to 28 days. Aboriginal patients accounted for 3.9% of events. A significantly lower proportion of Aboriginal than non-Aboriginal patients with IHD (50.2% vs 61.1%, p<0.001) and MI (69.6% vs 78.8%, p<0.001) received coronary angiography.

As shown in Table 1 (events-based characteristics) and Table 2 (person-based characteristics), Aboriginal patients admitted for IHD or MI events were significantly younger and more likely than their non-Aboriginal patients to be female, have lower SES and have comorbidities (pre-existing IHD, chronic pulmonary disease, diabetes, HF and kidney disease), but less likely to have private health insurance. Aboriginal patients had more events per person than non-Aboriginal: 25.5% vs 15.8% (p<0.001) respectively had two or more events (Table 2).

**Table 1:** **Demographic and clinical profile of IHD and MI events originating from WA metropolitan hospitals**

	**All IHD (n = 20,816 events)**			**MI only (n = 8,772 events)**		
	**Aboriginal**	**Non-Aboriginal**	**p value**	**Aboriginal**	**Non-Aboriginal**	**p value**
	**n = 817 events ** **for 552 patients**	**n = 19,999 events ** **for 16,091 patients**		**n = 309 events ** **for 253 patients**	**n = 8,463 events ** **for 7,880 patients**	
Age groups			<0.001			<0.001
25-34 years	17 (2.1)	85 (0.4)		7 (2.3)	46 (0.5)	
35-44 years	151 (18.5)	837 (4.2)		71 (23.0)	412 (4.9)	
45-54 years	257 (31.5)	2,798 (14.0)		94 (30.4)	1,273 (15.0)	
55-64 years	227 (27.8)	4,822 (24.1)		75 (24.3)	2,071 (24.5)	
65-74 years	127 (15.5)	5,235 (26.2)		44 (14.2)	2,091 (24.7)	
75-84 years	38 (4.7)	6,222 (31.1)		18 (5.8)	2,570 (30.4)	
Sex: Female	381 (46.6)	6,976 (34.9)	<0.001	129 (41.7)	2,532 (29.9)	<0.001
SES quartiles			<0.001			<0.001
1^st^ quartile^(a)^	436 (53.4)	5,240 (26.2)		161 (52.1)	2,015 (23.8)	
2^nd^ quartile	244 (29.9)	6,017 (30.1)		83 (26.9)	2,565 (30.3)	
3^rd^ quartile	95 (11.6)	4,488 (22.4)		46 (14.9)	1,933 (22.8)	
4^th^ quartile^(b)^	42 (5.1)	4,254 (21.3)		19 (6.1)	1,950 (23.0)	
Comorbidities						
Pre-existing IHD^(c)^	499 (61.1)	9,237 (46.2)	<0.001	146 (47.2)	2,487 (29.4)	<0.001
Chronic pulmonary disease	192 (23.5)	2,926 (14.6)	<0.001	58 (18.8)	1,036 (12.2)	0.001
Diabetes	463 (56.7)	5,790 (29.0)	<0.001	173 (56.0)	2,369 (28.0)	<0.001
HF	241 (29.5)	3,768 (18.4)	<0.001	101 (32.7)	1,767 (20.9)	<0.001
Kidney disease	180 (22.0)	2,144 (10.7)	<0.001	80 (25.9)	950 (11.2)	<0.001
Coronary angiography in the last year	134 (16.4)	2,447 (12.2)	<0.001	34 (11.0)	431 (5.1)	<0.001
Private health insurance (%)	61 (7.5)	7,277 (36.4)	<0.001	23 (7.4)	3,186 (37.6)	<0.001
IHD category (%)			0.04			
MI	309 (37.8)	8,463 (42.3)				
Unstable angina	315 (38.6)	7,042 (35.2)				
Other IHD	193 (23.6)	4,494 (22.5)				
MI subtype						0.08
Transmural				87 (28.2)	2,792 (33.0)	
Subendocardial/other				222 (71.8)	5,671 (67.0)	
Hospital at initial admission			<0.001			<0.001
Metropolitan tertiary	637 (78.0)	12,992 (65.0)		264 (85.4)	6,292 (74.3)	
Metropolitan non-tertiary^(d)^	103 (12.6)	1,678 (8.4)		11 (3.6)	237 (2.8)	
Private	77 (9.4)	5,329 (26.6)		34 (11.0)	1,934 (22.9)	

Statistical significance determined using chi-square tests. HF = heart failure; IHD = ischaemic heart disease; MI = myocardial infarction; SES = socio-economic status.

^(a)^most disadvantaged; ^(b)^least disadvantaged; ^(c)^excluding incident IHD; ^(d)^excluding private hospitals.

**Table 2:** **Demographic and clinical profile of IHD and MI patients at their first event**

	**All IHD (n = 16,643 patients)**			**MI only (n = 8,133 patients)**		
	**Aboriginal**	**Non-Aboriginal**	**p value**	**Aboriginal**	**Non-Aboriginal**	**p value**
	**n = 552 patients**	**n = 16,091 patients**		**n = 253 patients**	**n = 7,880 patients**	
Number of events			<0.001			<0.001
1	411 (74.5)	13,542 (84.2)		209 (82.6)	7,399 (93.9)	
2	84 (15.2)	1,822 (11.3)		32 (12.6)	399 (5.1)	
>2	57 (10.3)	727 (4.5)		12 (4.7)	82 (1.0)	
Age groups			<0.001			<0.001
25-34 years	16 (2.9)	79 (0.5)		7 (2.8)	46 (0.6)	
35-44 years	113 (20.5)	754 (4.7)		60 (23.7)	404 (5.1)	
45-54 years	155 (28.1)	2,377 (14.8)		70 (27.7)	1,222 (15.5)	
55-64 years	155 (28.1)	4,019 (25.0)		64 (25.3)	1,970 (25.0)	
65-74 years	81 (14.7)	4,219 (26.2)		35 (13.8)	1,962 (24.9)	
75-84 years	32 (5.8)	4,643 (28.9)		17 (6.7)	2,276 (28.9)	
Sex: Female	270 (48.9)	5,628 (35.0)	<0.001	103 (40.7)	2,332 (29.6)	<0.001
SES quartiles			<0.001			<0.001
1^st^ quartile^(a)^	289 (52.4)	4,000 (24.9)		129 (51.0)	1,845 (23.4)	
2^nd^ quartile	169 (30.6)	4,779 (29.7)		67 (26.5)	2,367 (30.0)	
3^rd^ quartile	62 (11.2)	3,651 (22.7)		40 (15.8)	1,807 (22.9)	
4^th^ quartile^(b)^	32 (5.8)	3,661 (22.8)		17 (6.7)	1,861 (23.6)	
Coronary angiography in the last year	33 (6.0)	927 (5.8)	0.83	12 (4.7)	253 (3.2)	0.18
Comorbidities						
Pre-existing IHD^(c)^	234 (42.4)	5,329 (33.1)	<0.001	90 (35.6)	1,904 (24.2)	<0.001
Chronic pulmonary disease	123 (22.3)	2,071 (12.9)	<0.001	45 (17.8)	914 (11.6)	0.003
Diabetes	293 (53.1)	4,179 (26.0)	<0.001	131 (51.8)	2,086 (26.5)	<0.001
HF	131 (23.7)	2,461 (15.3)	<0.001	72 (28.5)	1,465 (18.6)	<0.001
Kidney disease	98 (17.8)	1,302 (8.1)	<0.001	56 (22.1)	744 (9.4)	<0.001
Hospital at initial admission			<0.001			<0.001
Metropolitan tertiary	428 (77.5)	10,435 (64.8)		216 (85.4)	5,875 (74.6)	
Metropolitan non-tertiary^(d)^	69 (12.5)	1,272 (7.9)		8 (3.2)	215 (2.7)	
Private	55 (10.0)	4,384 (27.2)		29 (11.5)	1,790 (22.7)	

Statistical significance determined using chi-square tests.

HF = heart failure; IHD = ischaemic heart disease; MI = myocardial infarction; SES = socio-economic status.

^(a)^most disadvantaged; ^(b)^least disadvantaged; ^(c)^excluding incident IHD; ^(d)^excluding private hospitals.

### Aboriginal disparity in receipt of coronary angiography

After adjusting for age and sex, Aboriginal people with IHD were 23% less likely than non-Aboriginal people to receive coronary angiography (Model 1: RR_IHD_ 0.77, 95% CI 0.72-0.83) (Table 3). This disparity was diminished after progressive adjustments for SES (Model 2: RR_IHD_ 0.80, 95% CI 0.75-0.86), private health insurance (Model 3: RR_IHD_ 0.84, 95% CI 0.79-0.90) and IHD category and coronary angiography in the last year (Model 4: RR_IHD_ 0.87, 95% CI 0.82-0.93). Adjustment for individual comorbidities (Models 5a-e), especially HF (Model 5d: RR_IHD_ 0.91, 95% CI 0.86-0.97) and kidney disease (Model 5e: RR_IHD_ 0.91, 95% CI 0.85-0.97) substantially reduced the gap to 9% less likely. The disparity was not significant in the full model (Model 6: RR_IHD_ 0.95, 95% CI 0.89-1.01) which included all five comorbidities together. A similar pattern of reduction in the investigation gap was seen in the MI patient subgroup (Table 3). Much of the disparity was explained by comorbidities, especially kidney disease (Model 5e: RR_MI_ 0.91, 95% CI 0.85-0.97). There was no statistically significant difference in receipt of coronary angiography between Aboriginal and non-Aboriginal patients in the full model (Model 6: RR_MI_ 0.94, 95% CI 0.88-1.01).

**Table 3:** **Comparison of Aboriginal and non-Aboriginal probabilities of receipt of angiogram following IHD and MI events**

		**All IHD**		**MI only**	
**Aboriginal status = Yes**	**Description of model**	**RR (95** **%** **CI)**	**p value**	**RR (95** **%** **CI)**	**p value**
**Progressive adjustment:**					
Unadjusted		0.84 (0.78-0.90)	<0.0001	0.90 (0.83-0.96)	<0.01
Model 1	Age categories, sex	0.77 (0.72-0.83)	<0.0001	0.81 (0.75-0.87)	<0.0001
Model 2	Model 1 + SES	0.80 (0.75-0.86)	<0.0001	0.82 (0.76-0.88)	<0.0001
Model 3	Model 2 + private health insurance	0.84 (0.79-0.90)	<0.0001	0.84 (0.78-0.90)	<0.0001
Model 4	Model 3 + IHD category/MI subtype and angiography in the last year	0.87 (0.82-0.93)	<0.0001	0.86 (0.80-0.92)	<0.0001
Model 5 (comorbidities):					
Model 5a	Model 4 + pre-existing IHD	0.89 (0.84-0.95)	<0.001	0.88 (0.82-0.94)	<0.001
Model 5b	Model 4 + chronic pulmonary disease	0.89 (0.83-0.95)	<0.001	0.87 (0.81-0.94)	<0.001
Model 5c	Model 4 + diabetes	0.89 (0.83-0.95)	<0.001	0.88 (0.82-0.94)	<0.01
Model 5d	Model 4 + HF	0.91 (0.86-0.97)	<0.01	0.90 (0.84-0.97)	<0.01
Model 5e	Model 4 + kidney disease	0.91 (0.85-0.97)	<0.01	0.91 (0.85-0.97)	<0.01
Model 6	Model 4 + all five comorbidities (i.e. full model)	0.95 (0.89-1.01)	0.10	0.94 (0.88-1.01)	0.08
**Stratified by age** ^**(a)**^					
Aged 25–54 years:					
Model 1a	Model 1 but with age and age*age	0.83 (0.76-0.90)	<0.0001	0.85 (0.79-0.92)	<0.0001
Model 4a	Model 4 but with age and age*age	0.91 (0.85-0.99)	0.03	0.88 (0.82-0.95)	<0.001
Model 6a	Model 6 but with age and age*age	0.96 (0.89-1.04)	0.36	0.95 (0.88-1.02)	0.16
Aged 55–84 years:					
Model 1a	Model 1 but with age and age*age	0.70 (0.63-0.79)	<0.0001	0.73 (0.63-0.84)	<0.0001
Model 4a	Model 4 but with age and age*age	0.78 (0.70-0.87)	<0.0001	0.79 (0.69-0.91)	<0.001
Model 6a	Model 6 but with age and age*age	0.87 (0.79-0.97)	0.01	0.88 (0.77-0.99)	0.04
**Restricted to first event** ^**(a)**^					
Aged 25–54 years:					
Model 4a	Model 4 but with age and age*age	0.93 (0.85-1.01)	0.09	0.89 (0.83-0.97)	<0.01
Model 6a	Model 6 but with age and age*age	0.97 (0.89-1.05)	0.43	0.95 (0.88-1.02)	0.14
Aged 55–84 years:					
Model 4a	Model 4 but with age and age*age	0.80 (0.72-0.90)	<0.001	0.79 (0.69-0.91)	<0.001
Model 6a	Model 6 but with age and age*age	0.88 (0.79-0.99)	0.03	0.87 (0.77-0.99)	0.04
**Restricted to events without angiography in the previous year** ^**(a)**^					
Aged 25–54 years:					
Model 4a	Model 4 but with age and age*age	0.92 (0.85-0.99)	0.04	(b)	
Model 6a	Model 6 but with age and age*age	0.97 (0.89-1.04)	0.38	(b)	
Aged 55–84 years:					
Model 4a	Model 4 but with age and age*age	0.80 (0.72-0.89)	<0.0001	0.79 (0.69-0.91)	0.001
Model 6a	Model 6 but with age and age*age	0.88 (0.80-0.98)	0.02	0.88 (0.77-1.00)	0.05

Reference group is non-Aboriginal patients.

RR = risk ratio with reference group being non-Aboriginal patients; 95% CI = 95% confidence interval; HF = heart failure; IHD = ischaemic heart disease; MI = myocardial infarction; SES = socio-economic status.

^(a)^Models use age and age*age rather than age categories; ^(b)^unable to estimate.

A significant interaction was found for Aboriginal*age (p<0.05). Even when stratified by two age groups (25–54 years and 55–84 years), comorbidities accounted for much of the disparity in receiving coronary angiography (Model 4a versus 6a) (Table 3). Disparity between Aboriginal and non-Aboriginal events in the fully-adjusted model remained statistically significant in the older age group (55–84 years) for both IHD (Model 6a: RR_IHD_ 0.87, 95% CI 0.79-0.97) and MI (Model 6a: RR_MI_ 0.88, 95% CI 0.77-0.99) but not for either in the younger age group (25–54 years). As previous events and prior angiography may influence receipt of this procedure in future events, analyses were repeated with restriction to (i) first event only and (ii) events without prior angiography in the previous year, producing similar results for both all IHD and MI (Table 3).

### Individual characteristics associated with outcomes

In the full multivariable model for MI events, factors independently associated with a lower likelihood of receiving coronary angiography in the whole cohort were older age (65–84 years), being female, not having private health insurance, having coronary angiography in the previous year, and having chronic pulmonary disease, HF or kidney disease, while having transmural MI was associated with higher likelihood of receiving this procedure (Table 4). Factors in the younger group (25–54 years) associated with the likelihood of receiving angiography were similar to the whole cohort except that being female (RR_MI_ 1.00, 95% CI 0.97-1.04) and having pre-existing IHD (RR_MI_ 1.00, 95% CI 0.93-1.07) were not associated with a lower likelihood of receiving angiography, whereas having diabetes was independently associated with this outcome (RR_MI_ 0.95, 95% CI 0.91-0.99). In the older age group (55–84 years), apart from the lower likelihood of Aboriginal patients receiving angiography, estimates for all variables were similar to those for the whole cohort. SES was not independently associated with the outcome in either the whole cohort or the two age-groups.

**Table 4:** **Independent predictors for receipt of coronary angiography in MI events**

	**Receipt of coronary angiography in MI events**					
	**Whole cohort (n = 8,722 events)**		**Aged 25–54 years (n = 1,903 events)**		**Aged 55–84 years (n = 6,869 events)**	
	**RR (95%** **CI)**	**p value**	**RR (95%** **CI)**	**p value**	**RR (95%** **CI)**	**p value**
Aboriginal	0.94 (0.88-1.01)	0.08	0.95 (0.88-1.02)	0.16	0.88 (0.77-0.99)	0.04
Age groups						
25-34 years	1.01 (0.94-1.07)	0.87				
35-44 years	1.02 (0.99-1.04)	0.19				
45-54 years	1					
55-64 years	1.00 (0.98-1.02)	0.87				
65-74 years	0.97 (0.95-0.99)	0.01				
75-84 years	0.73 (0.71-0.76)	<0.0001				
Age			1.01 (0.98-1.03)	0.16	1.20 (1.17-1.24)	<0.0001
Age*age			1.00 (0.99-1.00)	0.51	0.99 (0.99-0.99)	<0.0001
Sex: Female	0.94 (0.92-0.96)	<0.0001	1.00 (0.97-1.04)	0.90	0.94 (0.91-0.96)	<0.0001
SES quartiles						
1^st^ quartile^(a)^	1		1		1	
2^nd^ quartile	1.01 (0.98-1.04)	0.44	0.99 (0.95-1.03)	0.50	1.02 (0.98-1.05)	0.37
3^rd^ quartile	1.00 (0.97-1.03)	0.83	0.99 (0.95-1.04)	0.74	1.00 (0.96-1.04)	0.99
4^th^ quartile^(b)^	0.99 (0.97-1.02)	0.70	1.00 (0.96-1.04)	0.95	0.99 (0.95-1.03)	0.54
No private health insurance	0.92 (0.90-0.93)	<0.01	0.97 (0.95-0.99)	0.01	0.91 (0.88-0.93)	<0.01
MI subtype^(c)^						
Transmural	1.10 (1.09-1.12)	<0.0001	1.04 (1.01-1.07)	<0.01	1.12 (1.10-1.15)	<0.0001
Subendocardial/other	1		1		1	
Coronary angiography in the previous year	0.74 (0.67-0.81)	<0.0001	0.77 (0.64-0.92)	<0.01	0.73 (0.66-0.82)	<0.0001
Pre-existing IHD^(d)^	0.95 (0.92-0.98)	<0.01	1.00 (0.93-1.07)	0.99	0.95 (0.92-0.99)	<0.01
Chronic pulmonary disease	0.89 (0.85-0.93)	<0.0001	0.85 (0.73-0.97)	0.02	0.89 (0.85-0.94)	<0.0001
Diabetes	0.99 (0.97-1.02)	0.51	0.95 (0.91-0.99)	0.01	1.00 (0.97-1.03)	0.78
HF	0.75 (0.72-0.79)	<0.0001	0.88 (0.80-0.97)	<0.01	0.75 (0.71-0.79)	<0.0001
Kidney disease	0.78 (0.73-0.81)	<0.0001	0.71 (0.57-0.89)	<0.01	0.79 (0.74-0.85)	<0.0001

^(a)^most disadvantaged; ^(b)^least disadvantaged; ^(c)^MI subtype was based on ICD-10-AM coding terminology; ^(d)^excluding incident IHD.

RR = Risk ratio; 95% CI = 95% confidence interval; IHD = ischaemic heart disease; HF = heart failure; MI = myocardial infarction; SES = socio-economic status.

### Sensitivity analyses

Table 5 shows the fully adjusted RRs (Model 6a) for (i) two alternative definitions of Aboriginal status (Aboriginal identification in at least 25% of hospital admissions or at first hospital admission for the event), (ii) 90-day events, (iii) different comorbidity look-back periods, (iv) hospital type at initial admission, and (v) inclusion of events where patients died within 28 days. The RRs for receipt of coronary angiography according to Aboriginal status in these analyses were very similar to those based on the original definitions of Aboriginal status, event duration, comorbidity look-back period, models that exclude hospital type and models that exclude events where patients died within 28 days.

**Table 5:** **Sensitivity analyses using different Aboriginal identification definitions, 90-day events, comorbidity look-back periods, hospital type and 28-day deaths**

	**All IHD**				**MI only**			
**Multivariate adjusted likelihood of Aboriginal patients receiving angiography**	**Aged 25–54 years**		**Aged 55–84 years**		**Aged 25–54 years**		**Aged 55–84 years**	
	**RR (95** **%** **CI)**	**p value**	**RR (95** **%** **CI)**	**p value**	**RR (95** **%** **CI)**	**p value**	**RR (95** **%** **CI)**	**p value**
(i) Definition of Aboriginal identification								
Any admission*	0.96 (0.89-1.04)	0.36	0.87 (0.79-0.97)	0.01	0.95 (0.88-1.02)	0.16	0.88 (0.77-0.99)	0.04
≥25% of admissions	0.95 (0.88-1.04)	0.27	0.89 (0.79-1.00)	0.05	0.94 (0.87-1.02)	0.13	0.87 (0.76-1.01)	0.07
First admission for the event	0.96 (0.88-1.05)	0.33	0.90 (0.80-1.02)	0.09	0.93 (0.86-1.01)	0.10	0.87 (0.75-1.01)	0.08
(ii) Definition of event duration								
28-day event (Aboriginal any admission)*	0.96 (0.89-1.04)	0.36	0.87 (0.79-0.97)	0.01	0.95 (0.88-1.02)	0.16	0.88 (0.77-0.99)	0.04
90-day event (Aboriginal any admission)	0.94 (0.87-1.01)	0.10	0.87 (0.79-0.96)	<0.01	0.96 (0.89-1.02)	0.19	0.87 (0.77-0.98)	0.02
(iii) Different comorbidity look-back periods								
1-year	0.95 (0.88-1.03)	0.18	0.84 (0.75-0.93)	0.001	0.94 (0.88-1.01)	0.12	0.86 (0.75-0.97)	0.002
2-year	0.96 (0.89-1.04)	0.29	0.85 (0.76-0.94)	0.002	0.95 (0.88-1.02)	0.17	0.86 (0.76-0.98)	0.02
5-year	0.96 (0.89-1.04)	0.35	0.86 (0.77-0.95)	0.003	0.95 (0.88-1.02)	0.17	0.87 (0.77-0.99)	0.03
10-year	0.96 (0.89-1.04)	0.36	0.86 (0.77-0.95)	0.003	0.95 (0.88-1.02)	0.14	0.87 (0.76-0.98)	0.03
15-year*	0.96 (0.89-1.04)	0.36	0.87 (0.79-0.97)	0.01	0.95 (0.88-1.02)	0.16	0.88 (0.77-0.99)	0.04
(iv) Model 6a + hospital type at initial admission	0.96 (0.88-1.04)	0.31	0.88 (0.80-0.98)	0.02	0.95 (0.88-1.02)	0.15	0.88 (0.77-0.99)	0.04
(v) Model 6a with inclusion of events where patients died within 28 days	0.97 (0.90-1.05)	0.46	0.88 (0.79-0.97)	0.01	0.96 (0.89-1.03)	0.25	0.88 (0.78-1.00)	0.06

For each sensitivity analysis, ‘*’ represents the original model (Model 6a from Table 3) which has been adjusted for age, age*age, sex, SES, private health insurance, IHD category (for IHD)/MI subtype (for MI), coronary angiography performed in the last year, 15-year histories of IHD, chronic pulmonary disease, diabetes, HF and kidney disease. Hospital type at initial admission is classified as metropolitan tertiary, metropolitan non-tertiary (excluding private hospital) or private. HF = heart failure; IHD = ischaemic heart disease; MI = myocardial infarction; RR = risk ratio; 95% CI = 95% confidence interval.

## Discussion

This study expands on other Australian research [4-7] investigating Aboriginal disparities in receipt of coronary angiography by restricting the analysis to metropolitan patients, and contributes to the sparse data on the health of Aboriginal people who live in metropolitan areas [13]. In this study of 20,816 acute IHD events (average 11 events/day) in metropolitan WA from 2005 to 2009, the distinctive demographic characteristics (younger, greater female representation, less likely to have private health insurance), higher prevalence of comorbidities and over-representation of Aboriginal patients were consistent with those reported previously [4,5,7,27]. When the differing socio-demographic and clinical profiles were taken into account by multivariate adjustment, a reduced likelihood of Aboriginal people receiving coronary angiography in comparison with non-Aboriginal people was only evident among older patients (aged 55–84 years). Higher comorbidities among Aboriginal people contributed substantially to the outcome disparities. The disparities for all IHD events and the MI subgroup were similar, so our discussion generally focuses on MI events unless comparison is made to other reported IHD findings.

The proportion of MI patients receiving coronary angiography in our study is slightly higher than that documented in the 2012 SNAPSHOT ACS study (78% vs 71%) conducted throughout Australia and New Zealand [28] and those of Randall *et al.* in New South Wales, Australia [5]. This likely reflects our more stringent case selection (MI in principal diagnosis field only) and our exclusive metropolitan focus. In our full multivariable model, the findings that older people, women and those without private health insurance were less likely to receive coronary procedures are consistent with those of previous studies [3,5,9,29,30]. SES was not associated with receiving angiography which may reflect SEIFA being an ecological measure of SES.

In relation to Aboriginal status, the results of our full model are consistent with Randall’s study [5], in which disparity in receiving coronary angiography was found for Aboriginal versus non-Aboriginal people after adjustment for age, sex, admission year, MI subtype and admitting hospital (adjusted hazard ratio [AHR] 0.81, 95% CI 0.74-0.88), and was largely explained by the higher burden of comorbidities, substance abuse and private health insurance among Aboriginal people (AHR 0.94, 95% CI 0.87-1.03). Randall found similar results for CARP (coronary angiography is a precursor to CARP), in that there was a large disparity between Aboriginal and non-Aboriginal people after adjusting for age, sex, year and MI subtype (AHR 0.63, 95% CI 0.57-0.70) and no significant disparity remaining after adjusting for hospital of admission, comorbidities, substance abuse and private health insurance (AHR 0.96, 95% CI 0.87-1.07) [5]. Similarly, a WA study using the Perth Aboriginal Atherosclerosis Risk Study cohort of metropolitan Aboriginal people found that CARPs for IHD were provided with equal frequency for Aboriginal people and age- and sex-matched non-Aboriginal people [31].

As there was an interaction between Aboriginal status and age, we modelled age by dichotomous stratification concomitantly with multivariate adjustment by age and age-squared as continuous variables. In doing so, we added to the findings of Randall [5], documenting that the disparity in receiving angiography was significant in the older Aboriginal patients (aged 55–84 years) but not in younger patients (aged 25–54 years), after adjusting for all other measured confounders. These findings are consistent with those of an Australian report which found that the largest differences in receipt of angiography between Aboriginal and non-Aboriginal IHD patients were among the 55–64 and 65–74 year age groups: in both, Aboriginal patients were half as likely to receive the investigation, compared with 90%, 70% and 60% for the 25–34, 35–44 and 45–54 year age groups respectively [32]. The disparity among older Aboriginal patients in our study is less than that in the report (13% vs 50% respectively) probably because in the latter, receipt of angiography during a single admission was determined from unlinked data, whereas we were able to identify angiography in any admission during a 28-day period. Furthermore, our study examined IHD events defined by an initial emergency admission, whereas the report included all IHD admissions, including elective admissions in which clinician discretion and patient preference may play a larger role. From administrative data, we are unable to elucidate the reasons for the greater disparity for Aboriginal patients in the older age group. In general, elderly patients with MI are known to be managed more conservatively than their younger counterparts, on the basis of anticipated poorer outcomes partly attributable to comorbidities, but the basis for this conservatism has been questioned [33,34]. Eligibility for reperfusion declines with age, and yet elderly patients are less likely to receive reperfusion even if eligible [35]. Explanations for the disproportionate reduction in likelihood of angiography among older Aboriginal patients could include confounding by unmeasured clinical characteristics as well as clinicians’ ‘therapeutic nihilism’ in relation to older Aboriginal patients or a higher level of patient refusal compared to younger Aboriginal patients.

Regardless of age, the greatest contributors to the reduced likelihood of having an angiogram were comorbidities, especially HF and kidney disease, and to a lesser extent chronic pulmonary disease. HF and pre-existing kidney disease, which are substantially over-represented in Aboriginal patients, are important risk factors for contrast-induced acute kidney injury in the setting of coronary angiography [36] and hence the probability of having this procedure is substantially lower amongst patients with these comorbidities [3]. It remains to be determined if the lower use of coronary angiography among patients with these pre-existing comorbidities is attributable to physician reluctance to perform the procedure because of the perceived risk [37], ‘therapeutic nihilism’ in relation to such patients, or whether a different clinical presentation of acute MI among such patients influences decision making by physicians [36]. The decision to perform coronary angiography in patients with these comorbid conditions remains vexed: the poorer prognosis of IHD associated with these comorbidities augments the anticipated absolute benefit from definitive interventions (i.e. CARPs) [38] yet angiographic contrast-induced acute kidney injury is clearly associated with prolonged hospitalisation and an increased likelihood of renal impairment, cardiovascular events and death. However, mortality attributed to this adverse reaction to contrast may have been widely over-reported in unadjusted studies, being strongly confounded by baseline clinical status [39].

The strength of this study was its use of person-linked hospital and mortality data with state-wide coverage, allowing us to follow patients throughout their 28-day events. The use of 28-day events was based on previous studies and ICD-10 coding standards [40-42]; repeating the analysis for 90-day events produced similar results, indicating that the relative risks are robust with respect to the definition of an event. Although Aboriginal status is under-reported in administrative data, it is improving [43]. Our sensitivity analyses with three methods of Aboriginal identification produced similar results indicating the findings are robust with respect to Aboriginal identification. Similarly, our results were robust to previous events and receipt of coronary angiography in the last year. Possible under-identification of comorbidities did not influence our findings as the adjusted RRs for Aboriginal status was similar with 1-, 2-, 5-, 10- and 15-year look-back periods. However, there are limitations of routinely collected administrative data. For example, delineation of the reasons for the observed disparities is limited by the absence of detailed data on clinical characterisation of events (e.g. time-to-presentation, electrocardiographic/biomarker findings) in relation to evidence-based guidelines and standard practice for performing angiography, as well as the absence of information on patient preference in relation to invasive investigations. The use of SEIFA scores as an ecological measure of SES obscures heterogeneity of household SES status within a collection district–many collection districts comprise socio-economically disparate subgroups (notably including Aboriginal people).

## Conclusion

Evidence-based risk versus benefit considerations justify a somewhat more conservative approach to acute IHD in older patients, ramifying in a lower likelihood of receiving coronary angiography. However, departure from clinical guidelines in actual practice resulting in under-management of older patients is a well described problem [34,35]. The disproportionate under-management of older Aboriginal IHD patients requires further exploration through clinical and qualitative studies. Regardless of age, much of the disparity experienced by metropolitan Aboriginal patients with IHD in receiving angiography is accounted for by their high prevalence of comorbidities. Our results echo those of the Australian and New Zealand SNAPSHOT ACS study [28] in that the burden of comorbidities accentuates the challenges faced in applying evidence-based guidelines among patients in this context. The constellation of health problems interfacing with IHD (including chronic pulmonary disease, diabetes, HF and kidney disease) accounts for much of the disease burden and gap in life expectancy encountered by Aboriginal people [44,45]. If, as suggested by the results of the current study, the disparity faced by metropolitan Aboriginal patients in receiving coronary angiography for acute IHD is mediated substantially by comorbidities; the presence of these may be considered a ‘double-whammy’ for Aboriginal Australians, predisposing them to IHD while also adversely impacting their receipt of coronary procedures. Our findings of relatively modest disparities after adjustment for measurable influences should not be interpreted as a basis for complacency, but rather they highlight the need for intensified preventative activity and improved service delivery to Aboriginal people, addressing multimorbidity along with attention to the underlying social determinants of health.

## Abbreviations

ACS: Acute coronary syndrome
AHR: Adjusted hazards ratio
CARP: Coronary artery revascularisation procedure
HF: Heart failure
HMDC: Hospital morbidity data collection
ICD-10-AM: International classification of diseases Australian modification 10th revision
IHD: Ischaemic heart disease
MI: Myocardial infarction
RR: Risk ratio
SEIFA: Socio-economic indexes for areas
SES: Socio-economic status
WA: Western Australia
WADLS: Western Australian data linkage system
